# Partial nephrogenic diabetes insipidus caused by a novel *AQP2* variation impairing trafficking of the aquaporin-2 water channel

**DOI:** 10.1186/s12882-015-0213-3

**Published:** 2015-12-29

**Authors:** Pia Dollerup, Troels Møller Thomsen, Lene N. Nejsum, Mia Færch, Martin Österbrand, Niels Gregersen, Søren Rittig, Jane H. Christensen, Thomas J. Corydon

**Affiliations:** Department of Biomedicine, Aarhus University, Wilhelm Meyers Allé 4, 8000 Aarhus, Denmark; Department of Molecular Biology and Genetics and iNANO, Aarhus University, Aarhus, Denmark; Department of Pediatrics, Aarhus University Hospital, Aarhus, Denmark; Department of Pediatrics, Queen Silvia Children’s Hospital, Gothenburg, Sweden; Research Unit for Molecular Medicine, Department of Clinical Medicine, Aarhus University Hospital, Aarhus, Denmark

**Keywords:** Aquaporin 2, Diabetes insipidus, Congenital nephrogenic diabetes insipidus, Lentivirus, Cellular trafficking, Intracellular localization

## Abstract

**Background:**

Autosomal dominant inheritance of congenital nephrogenic diabetes insipidus (CNDI) is rare and usually caused by variations in the *AQP2* gene. We have investigated the genetic and molecular background underlying symptoms of diabetes insipidus (DI) in a Swedish family with autosomal dominant inheritance of the condition.

**Methods:**

The proband and her father were subjected to water deprivation testing and direct DNA sequencing of the coding regions of the *AQP2* and *AVP* genes. Madin-Darby canine kidney (MDCK) cells stably expressing AQP2 variant proteins were generated by lentiviral gene delivery. Localization of AQP2 variant proteins in the cells under stimulated and unstimulated conditions was analyzed by means of immunostaining and confocal laser scanning microscopy. Intracellular trafficking of AQP2 variant proteins was studied using transient expression of mutant dynamin2-K44A-GFP protein and AQP2 variant protein phosphorylation levels were assessed by Western blotting analysis.

**Results:**

Clinical and genetic data suggest that the proband and her father suffer from partial nephrogenic DI due to a variation (g.4807C > T) in the *AQP2* gene. The variation results in substitution of arginine-254 to tryptophan (p.R254W) in AQP2. Analysis of MDCK cells stably expressing AQP2 variant proteins revealed disabled phosphorylation, impaired trafficking and intracellular accumulation of AQP2-R254W protein. Notably, blocking of the endocytic pathway demonstrated impairment of AQP2-R254W to reach the cell surface.

**Conclusions:**

Partial CNDI in the Swedish family is caused by an *AQP2* variation that seems to disable the encoded AQP2-R254W protein to reach the subapical vesicle population as well as impairing its phosphorylation at S256. The AQP2-R254W protein is thus unable to reach the plasma membrane to facilitate AVP mediated urine concentration.

**Electronic supplementary material:**

The online version of this article (doi:10.1186/s12882-015-0213-3) contains supplementary material, which is available to authorized users.

## Background

Congenital nephrogenic diabetes insipidus (CNDI) is a disorder characterized by polyuria and polydipsia due to renal resistance to the antidiuretic hormone, arginine vasopressin (AVP). The majority of cases are caused by variations in the *AVPR2* gene encoding the renal V2 receptor or by autosomal recessively inherited variations in the *AQP2* gene encoding the collecting duct water channel aquaporin-2 (AQP2) [1]. AQP2 forms tetramers that are localized to sub-apical vesicles and the apical plasma membrane in kidney collecting duct principal cells [2, 3]. Upon AVP stimulation, AQP2 is phosphorylated at the C-terminal S256 by Protein Kinase A (PKA) [4], where after AQP2-containing subapical vesicles are inserted into the apical plasma membrane, facilitating AQP2 accumulation and thus, urine concentration. Evidence suggests that AQP2 constitutively shuttles between the plasma membrane and subapical vesicles [5], and that the life-time in the plasma membrane is determined by phosphorylation at S256 [6, 7]. It has been shown that AQP2-S256A, which mimics constitutively non-phosphorylated AQP2, accumulates in the plasma membrane if endocytosis is blocked by expressing dominant-negative dynamin-2 [8]. Thus, it seems that S256 phosphorylation shifts the balance of AQP2 recycling, facilitating AQP2 accumulation in the plasma membrane. In addition, phosphorylation at S256 on at least three out of four monomers in the AQP2 tetramer seems to be necessary for accumulation of AQP2 in the apical membrane [9–11].

Defective trafficking of AQP2 in response to AVP stimulation is suggested as the cause of the rare cases of dominantly inherited CNDI [12, 13] and dominant inherited variations affecting the PKA consensus site are suggested to disable phosphorylation at S256 [14, 15] and consequently, disrupt the kidneys urinary concentration ability.

Variant AQP2 monomers are further believed to have a dominant negative effect on wildtype (WT) monomers resulting in altered trafficking, missorting or retention in the Golgi apparatus [13, 16–18]. Of special note, the majority of variations causing dominantly inherited CNDI are associated with partial diabetes insipidus (DI), where affected subjects retain an ability to concentrate urine in response to high levels of AVP or desamino-8-D-arginine vasopressin (dDAVP). This has led to the suggestion that functional AQP2 tetramers either in the form of WT homo-tetramers or WT-variant hetero-tetramers are formed and reaches the apical plasma membrane [1, 12, 14, 15, 18].

In the present study, we identified a novel g.4807C > T variation in the *AQP2* gene in a Swedish family with dominant inheritance of CNDI. This variation causes substitution of arginine-254 to tryptophan (AQP2-R254W) in the C-terminal of AQP2. In order to substantiate that the variation identified is causal to CNDI in the family and investigate the underlying mechanism responsible, we further investigated the cellular handling of the AQP2 protein in Madin-Darby canine kidney (MDCK) cells stably expressing AQP2-R254W.

## Methods

### Clinical tests

The family history was recorded in order to determine the presence of symptoms of DI in a Swedish family and a water deprivation test (WDT) was performed according to normal clinical practice. Regarding the clinical tests The Regional Committee on Biomedical Research Ethics has agreed that ethical approval and consent to participate was not required. The parents of the patient granted written informed consent for publication of this report as the patient was underage.

### Gene sequencing

Genomic DNA was extracted from full blood using QIAamp DNA Blood Mini Kit (Qiagen Nordic, Copenhagen, Denmark). The coding regions of both the *AVP* and the *AQP2* gene was amplified by polymerase chain reaction and the amplified gene products was sequenced using BigDye Terminator v1.1 Cycle Sequencing Kit (Applied Biosystems, Foster City, CA). Sequencing products was analyzed using ABI Prism 3100 Avant Genetic Analyser (Applied Biosystems).

### Cell culture

Polarized Madin-Darby canine kidney (MDCK; catalog number CCL-34TM, American Type Culture Collection, Boras, Sweden) cells were used to resemble the collecting ducts *in vivo*. Cells were cultivated in tissue culture flasks (TPP, Techno Plastic Products AG, Trasadingen, Switzerland) in complete Dulbecco's Modified Eagle's Medium (DMEM) (Gibco, Invitrogen, Taastrup, Denmark) containing 0.29 mg/ml glutamine (Sigma-Aldrich, Broendby, Denmark), 0.06 mg/ml penicillin (Farma-Plus, Oslo, Norway), 0.1 mg/ml streptomycin (Sigma-Aldrich) and 10 % fetal calf serum (Sigma-Aldrich). The cells were maintained at 37 °C and 5 % CO_2_.

### Lentiviral production and transduction of MDCK cells

In order to produce MDCK cells stably expressing AQP2-protein, lentivirus (LV) vectors encoding AQP2-WT and AQP2-R254W were produced (see below). For comparison purposes we also generated LVs encoding AQP2-R254L [14]. The LV vector constructs contain a phosphoglycerate kinase (PGK) promoter, the *AQP2* cDNA (encoding WT, R254W or R254L proteins), an internal ribosome entry site sequence (IRES) and a sequence coding for resistance towards puromycin (puro), enabling selection of the transduced cells. A schematic presentation of the LV construct is depicted in Fig. 3a.

Initially, WT AQP2 cDNA (ImaGenes, Berlin, Germany) was cloned into pcDNA3.1 vector (Invitrogen) using *Bgl*II*/Xho*I and *Bam*HI*/Xho*I, respectively (New England Biolabs). The desired mutation was introduced using Quick Change Site-Directed Mutagenesis Kit (Stratagene, AH Diagnostics A/S, Aarhus, Denmark). To generate *AQP2* c.760C > T cDNA (corresponding to *AQP2* g.4807C > T), encoding R254W mutant protein, sense primer 5’-gaggtgcgaggtggcagtcggtgg-3’ and antisense primer 5’-ccaccgactgccaccgtcgcacctc-3’ were used. Underlined bases mark the targets for the mutagenesis. The AQP2-R254L (GenScript, New Jersey, USA) was likewise cloned into pcDNA3.1 vector using *Bgl*II*/Xho*I and *Bam*HI*/Xho*I. The three *AQP2* cDNA sequences were subsequently excised from the corresponding pcDNA3.1 vectors using *Pme*I and *Xba*I and ligated into the *Pme*I and *Xba*I sites of the pCCL-PGK-Puro plasmid [19]. The resulting LV vectors were named pLV/PGK-AQP2-WT, pLV/PGK-AQP2-R254W and pLV/PGK-AQP2-R254L, respectively. Plasmid DNA was purified using Plasmid*Plus* kits (Qiagen Nordic). Restriction enzyme analysis and DNA sequencing verified all constructs.

LV vectors were generated as previously described [19, 20]. In brief: On day 1, 1x10^7^ human embryonic kidney (HEK-293 T; catalog number CRL-3216, American Type Culture Collection) cells were seeded in p15 dishes (Greiner, Sigma-Aldrich) containing complete DMEM (see above). On day 2, a transfection mix of 7.3 μg pRSV-Rev, 9.1 μg pMD.2G, 31.5 μg pMDIg/p-RRE, 31.5 μg AQP2 plasmid and 1 μg plasmid DNA encoding enhanced green fluorescent protein (GFP) were prepared for each dish, and the cells were transfected using calcium phosphate transfection. On day 3, the medium was replaced with fresh complete DMEM. On day 4, the supernatant with virus were harvested and filtered through a 0.45 μm filter (Millipore A/S, Hellerup, Denmark), and thereafter purified by ultracentrifugation through a sucrose solution at 25,000 rpm for 2 h. The virus pellet were resuspended in PBS and stored at -70 °C. Concentrations of the produced lentivirus were determined by measuring HIV-1 p24 by enzyme-linked immunoassay (Zeptometrix, Buffalo, NY, USA).

Approximately 8x10^4^ MDCK cells were seeded in 6-well-plates (TPP) the day before transduction. The transduction was performed with 40 ng p24/well. The LVs was added to complete DMEM medium containing 8 μg/mL polybrene (Sigma-Aldrich) and exchanged with the medium in the plates. Selection with 10 μg/mL puromycin (Invitrogen) started 72 h post-transduction. Wells containing untransduced MDCK cells were used as controls. Comparable fluorescent signals (Fig. 3) and amounts of total AQP2 protein (Fig. 5c) were identified in cells transduced with LV vectors encoding AQP2-WT, AQP2-R254W or AQP2-R254L showing that AQP2 is similarly expressed in the three cell lines. However, it should also be mentioned that intercellular variation in AQP2 expression, most likely representing copy number differences and/or diverse integration sites as each of the cell lines was obtained by pooling puromycin resistant cells, was observed following immunostaining and CLSM analysis.

### Immunostaining and confocal laser scanning microscopy

For investigation of the localization and trafficking of variant AQP2, immunostaining and confocal laser scanning microscopy (CLSM) experiments were performed. On day 1, MDCK cells were seeded at confluency on Transwell polycarbonate membranes (12 mm, 0.4 μm pore size, Costar, Sigma-Aldrich). After 24 h culture medium was changed and the cells were treated with the basal cAMP lowering drug indomethacin (10 μM) over night. On day 3, the cells were incubated in preheated PBS-ABC (PBS (Sigma-Aldrich) containing 1 mM CaCl_2_, 0.5 mM MgCl_2_) with (150 μM) or without forskolin for 1 h at 37°. Then cells were washed 3x5 min. in PBS-ABC. Thereafter the cells were fixed in 4 % paraformaldehyde (Lillies buffer, Buch & Holm, Herlev, Denmark) for 5 min. and washed 3x5 min. in PBS-ABC. Prior to immunostaining the cells were permeabilized with PBS solution containing 3 % BSA and 0.3 % Triton X-100 for 15 min. at room temperature. The Transwell membranes were then cut out from their plastic support and subjected to antibody solutions on parafilm.

Primary antibodies (polyclonal goat anti - AQP2 antibody, C-17, Santa Cruz Biotechnology, Dallas, Texas, USA, and polyclonal rabbit anti - ZO-1 antibody, Mid, Invitrogen) were diluted 1:100 in blocking buffer (BB, 0.5 % (w/v) bovine serum albumin in PBS-ABC) and the cells were incubated in the solution for 1 h at room temperature, followed by three washing steps of 5 min. in PBS. Secondary antibodies (Alexa Fluor® 488 donkey anti - goat, catalog number A-11055, and Alexa Fluor® 568 polyclonal donkey anti-rabbit, catalog number A-10042, both Molecular Probes, Invitrogen) were likewise diluted in BB (1:400), and the cells were incubated in the solution for 1 h at room temperature. Finally, the cells were washed 3x5 min. in PBS-ABC before incubated with 4',6-diamidino-2-phenylindole (DAPI) (Sigma-Aldrich) for 5 min. Finally, cells were washed 2x5 min. in PBS and carefully mounted on glass slides using Glycergel (Dako, Glostrup, Denmark) supplemented with 1,4-Diazabicyclo(2,2,2)octane (2.5 % w/v) (Sigma-Aldrich). Images of AQP2-WT, AQP2-R254W and AQP2-R254L were captured on a CLSM (LSM 710, Zeiss, Jena, Germany) by using a 488 nm line of a multiline argon laser (detection of Alexa-488), the 405 nm line of a 405-430 nm diode laser (detection of DAPI), and an 63 × oil-immersion objective with a NA of 1.4.

### Transfection with plasmid DNA encoding dynamin2-, Rab7- and Rab9-GFP fusion proteins

Transfections experiments were performed using Lipofectamine 2000 (Invitrogen) following the manufacturer’s recommendations. MDCK-AQP2-WT, MDCK-AQP2-R254W and in some cases MDCK-AQP2-R254L cells (as indicated) were seeded on Transwell polycarbonate membranes (12 mm, Costar) covered by DMEM without fetal calf serum.

Endocytosis of AQP2 was investigated by transfecting the cells with 0.4 μg Dyn2-WT-GFP or 0.4 μg Dyn2-K44A-GFP plasmid DNA, encoding WT or mutant dynamin proteins C-terminally fused to GFP, respectively (Addgene, Cambridge, MA, USA). Upon expression of the Dyn2-K44A-GFP protein, the dynamin-2-dependent endocytic pathway is blocked. For localization of late endosomes, MDCK cells expressing AQP2-WT, AQP2-R254W or AQP2-R254L were transfected with 0.4 μg Rab7-GFP and 0.4 μg Rab9-GFP, respectively. The Rab7-GFP and Rab9-GFP plasmids encodes GFP-tagged variants of Rab7 [21] and Rab9 [22] involved in retrograde transport as well as transport between late endosomes and the trans-Golgi network.

Medium was replaced 6 h and 24 h post-transfection. Two days after transfection the cells were fixed in 4 % paraformaldehyde (Buch & Holm) for 5 min. Prior to immunostaining of AQP2 the cells were permeabilized with PBS solution with 3 % BSA and 0.3 % Triton X100 for 15 min. A polyclonal goat anti - AQP2 antibody (C-17, Santa Cruz Biotechnology) was used as primary antibody in a 1:100 dilution. Alexa Fluor® 568 donkey anti - goat, was used as secondary antibody (1:400, catalog number A-11057, Molecular Probes). Visualization was performed by using a 488 nm line of a multiline argon laser (detection of GFP), the 563 nm line of a helium-neon laser (detection Alexa-568), the 405 nm line of a 405-430 nm diode laser (detection of DAPI), and an 63× oil-immersion objective with a NA of 1.4 on a LSM 710 confocal laser scanning microscopy (Zeiss).

### Identification of phosphorylated AQP2 by Western blotting analysis

On day 1, transduced MDCK-cells were seeded in T25 culture flasks (TPP) at approx. 20 % confluency. On day 2, the medium was exchanged with fresh medium containing indomethacin (10 μM). Day 3: The media was removed and exchanged with preheated PBS-ABC with or without forskolin for 1 h (150 μM, Sigma-Aldrich). The cells were then harvested and centrifuged for 3 min. at 220 g and the supernatant discharged. Lysis buffer (50 mM Tris-HCl, 5 mM EDTA, 1 mM DTE, 10 μg/mL aprotinin, 10 mg/mL soyabean trypsin inhibitor, 1 tablet Complete mini and 1 % Triton X-100) were added to the pellet and incubated on ice for 1 h [23]. The samples were then centrifuged shortly at 10,000 g and protein concentration was measured on the supernatant fraction (Bradford Protein Assay, BioRad, Copenhagen, Denmark). Western blotting analysis was performed as previously described [23]. In brief, 10 μg total protein was loaded on a 12 % polyacrylamide gel (Criterion TGX gel, BioRad) together with a Precision Plus Protein All Blue Standards marker (BioRad) followed by transfer to a PVDF midi membrane (BioRad) using the Trans-Blot TurboTransfer system (BioRad). The membrane was subsequently cut in three parts before immunolabeling was performed using individual antibodies targeting AQP2, phosphorylated AQP2 (p-AQP2) [4, 11] and ß-actin. For detection of AQP2 a primary polyclonal goat anti - AQP2 antibody (1:500, C-17, Santa Cruz Biotechnology) was used. For detection of phosphorylated AQP2 a phosphorylation sensitive rabbit p-AQP2 antibody (1:1,000, KO307, kindly provided by Professor Søren Nielsen, Department of Biomedicine, Aarhus University, Denmark) was applied. As a loading control, polyclonal rabbit antibodies against ß-actin (1:10,000, catalog number 8227-50, Abcam, Cambrigde, MA, USA) were used. Detection was performed using secondary horseradish peroxidase (HRP) conjugated polyclonal rabbit anti-goat and polyclonal goat anti-rabbit HRP conjugated antibodies (1:10.000, catalog numbers P0449 and P0448, Dako). All antibodies were diluted in TBS-T (0.9 % NaCl, 20 mM Tris-HCl added 0.1 % Tween 20 (Sigma)). For visualization SuperSignal West Dura Extended Duration Substrate (Thermo Scientific, Waltham, MA, USA) and ImageQuant Las4000 digital imaging system (GE Healthcare, Cleveland, OH, USA) were used.

The cellular experiments were replicated minimum three times unless otherwise stated.

## Results

### Clinical data indicate partial CNDI

**Fig. 1:**
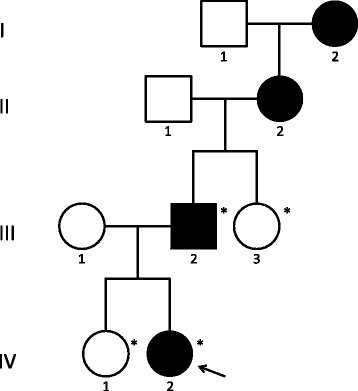
Pedigree showing a Swedish family with autosomal dominant inheritance of congenital nephrogenic diabetes insipidus. The affected individuals are illustrated with filled symbols. Asterisks indicate the members of the family who were genetically tested. The proband is marked with an arrow

A Swedish family including four family members with symptoms of DI was investigated (Fig. 1). The proband (subject IV-2) was first admitted to hospital at nine months of age, presenting with polydipsia, polyuria and signs of failure to thrive. The extremely high fluid intake of 200 ml/kg bw/24 h of the proband reported by the parents and very low basal urine osmolality on two occasions strongly suggested the diagnosis of DI. Testing of the proband upon admission revealed a low spot urine osmolality of 122 mOsm/kg and a normal plasma osmolality of 278 mOsm/kg. Treatment of the proband with 2.5 μg intranasal dDAVP once daily led to no improvement of thirst and urine output. The father of the proband was initially suspected to suffer from familial neurohypophyseal DI (FNDI), as he was responding to dDAVP treatment.

A water deprivation test (WDT) was performed in the proband (Table 1) at the age of 21 months. Prior to WDT her basal urine osmolality was 32 mOsm/kg, while plasma sodium (137 mmol/L) and osmolality (286 mOsm/kg) was within control range. Plasma AVP was slightly elevated (2.3 pg/mL) considering the plasma osmolality level. After six hours of thirst the urine osmolality increased to 268 mOsm/kg, plasma sodium to 139 mmol/L, plasma osmolality to 295 mOsm/kg and plasma AVP displayed a 2.4 fold increase (from 1.3 to 3.1 pg/mL) and body weight was reduced by 3.7 % (Table 1). The father was likewise subjected to a WDT (Table 2). Prior to water restriction his urine osmolality was 126 mOsm/kg and plasma osmolality was 290 mOsm/kg. After WDT the osmolality reached 613 mOsm/kg and plasma osmolality 303 mOsm/kg, respectively. By increasing the dose of dDAVP given to the proband gradually to 40 μg three times daily, symptoms of DI were alleviated for 4-5 h with subsequent improvement of sleep and well-being.

**Table 1:** Water deprivation test in subject IV-2

	Body Mass (gram)	Urine Osmolality (mOsm/kg)	Plasma Osmolality (mOsm/kg)	Plasma Sodium (mmol/L)	Plasma AVP (pg/mL)
Night before WDT	9120	44	292	133	2.3
Morning before WDT	8930	32	286	137	1.3
During WDT		54			3.2
After WDT	8600	268	295	139	3.1

*WDT* water deprivation test

**Table 2:** Water deprivation test in subject III-2

	Urine Osmolality (mOsm/kg)	Plasma Osmolality (mOsm/kg)
Before WDT	126	290
After WDT	613	303

*WDT* water deprivation test

Since the plasma osmolality of the proband did not exceed 300 mOsm/L before termination of the WDT test and no additional clinical examinations have been achievable since then, the differential diagnosis remains unclear. The relatively high level of plasma AVP under basal conditions (2.3 pmol/mL) seen in the proband together with a normal AVP response to dehydration as well as only partial alleviation of symptoms using high doses of dDAVP, indicates segregation of a partial *nephrogenic* DI phenotype (CNDI) in the family.

### DNA sequence analysis revealed a novel variation in the AQP2 gene

**Fig. 2:**
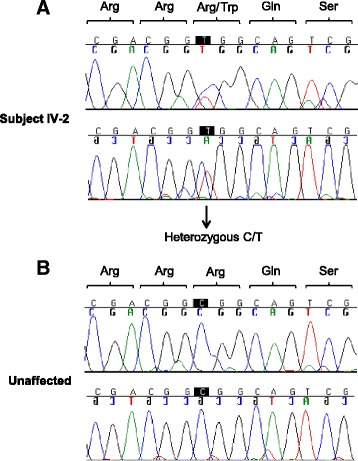
Identification of a novel *AQP2* variation in affected individuals. Direct DNA sequence analysis of the coding regions of the *AQP2* gene in: (**a**) subject IV-2 and (**b**) an unaffected control subject. Data from both the sense and anti-sense strands corresponding to genomic region g.4801–4815 within exon 4 of the *AQP2* gene are shown (numbers refer to the genomic DNA sequence of the *AQP2* gene, GenBank accession number P41181). The affected subject IV-2 is heterozygous for a single base substitution (g.4807C > T) resulting in a double peak in the electropherogram (arrow). The amino acid residues encoded by these sequences are shown above using three-letter abbreviations

In order to confirm the diagnosis and specifically identify its genetic cause, molecular genetic analysis was performed in the two affected subjects as well as in two unaffected family members (subject III-3 and IV-1, Fig. 1). DNA sequencing analysis of the coding regions of the *AQP2* gene in the proband (subject IV-2) revealed a novel g.4807C > T variation in exon 4 in one allele of the gene (number refer to the genomic DNA sequence of the *AQP2* gene, GenBank accession number P41181) (Fig. 2a). The identified variation is predicted to change codon 254 of the AQP2 mRNA from CGG to UGG resulting in an arginine to tryptophan substitution (p.R254W) in the C-terminal of AQP2. No other sequence variations were found in the coding regions of the *AQP2* gene. An identical genotype was identified in the probands farther (subject III-2). The unaffected subjects (III-3 and IV-1) were both homozygous for the normal allele. Similarly, an unrelated control subject (unaffected) was homozygous for the normal allele (Fig. 2b). Common variations at this position were neither identified by sequence analysis of 100 alleles from healthy Danish individuals nor in the 1000 Genomes database (http://www.1000genomes.org/). DNA sequencing analysis of the coding regions of the *AVP* gene in the proband did not reveal any variations. Overall, these data strongly indicate that the affected subjects suffer from partial CNDI due to heterozygosity for the p.R254W substitution in AQP2.

### Verification of transduction capacity of the LV vector in MDCK cells

**Fig. 3:**
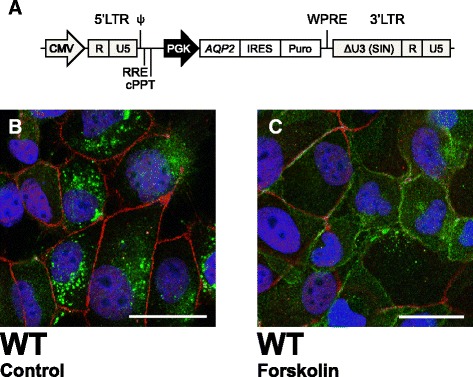
Localization of AQP2-WT in MDCK cells under unstimulated and forskolin stimulated conditions. MDCK cells transduced with LVs containing the *AQP2-WT* gene for stably expression of AQP2-WT were incubated with 150 μM forskolin prior to immunostaining for AQP2 and tight junctions, ZO-1. The cells were then subjected to CLSM analysis. (**a**) Schematic representation of the utilized LV expression cassette. The LV transfer vector incorporates a PGK promoter that drives the expression of a single mRNA encoding both AQP2 and puromycin. (**b**) Unstimulated MDCK cells expressing AQP2-WT. (**c**) AQP2-WT in MDCK cells after forskolin stimulation. Scale bars = 20 μm. CMV, cytomegalovirus; cPPT, central polypurine tract; LTR, long terminal repeat; PGK, phosphoglycerate kinase 1 promoter; ψ, packaging signal; RRE, Rev-responsive element; U3 (SIN), Self-inactivating deletion in the U3 region; WPRE, woodchuck hepatitis virus post-transcriptional regulatory element

The identified variation results in an amino acid substitution in the C-terminal tail of the protein, suggesting altered trafficking of AQP2 [24]. Thus, to investigate the cellular handling of AQP2-R254W we designed and purified lentivirus (LV) vectors containing the *AQP2* c.760C > T cDNA or the *AQP2* WT gene driven by a phosphoglycerate kinase (PGK) promoter (Fig. 3a). The resulting LV vector constructs encodes AQP2-R254W or AQP2-WT proteins. Other variations in codon 254 of the AQP2 mRNA, likewise resulting in amino-acid changes such as the AQP2-Arg254Leu (AQP2-R254L) substitution, have been described to affect Ser256 phosphorylation and intracellular trafficking of AQP2 [14, 15]. Thus, LVs expressing AQP2-R254L were included as a positive control for impaired phosphorylation and defective intracellular trafficking [14]. To confirm functionality and the capacity of the LV vector to transduce MDCK cells, we initially delivered vectors encoding AQP2-WT and collected data by immunostaining and CLSM analysis (Fig. 3b and c). The cells were stained with an antibody directed against AQP2 as well as an antibody labeling tight junctions (ZO-1), the boundary between apical and basolateral membrane domains. Sustained expression of AQP2-WT protein, solely displaying an intracellular staining pattern without any AQP2-specific staining of the plasma membrane, was evident in unstimulated cells (Fig. 3b, Control). Upon stimulation with forskolin, an adenylyl cyclase activator that mimics the action of AVP by increasing the intracellular level of cAMP, the localization of AQP2-WT protein was clearly shifted from cytoplasmic vesicles to the cell membrane (compare Fig. 3b and c).

### Impaired intracellular trafficking of AQP2-R254W

**Fig. 4:**
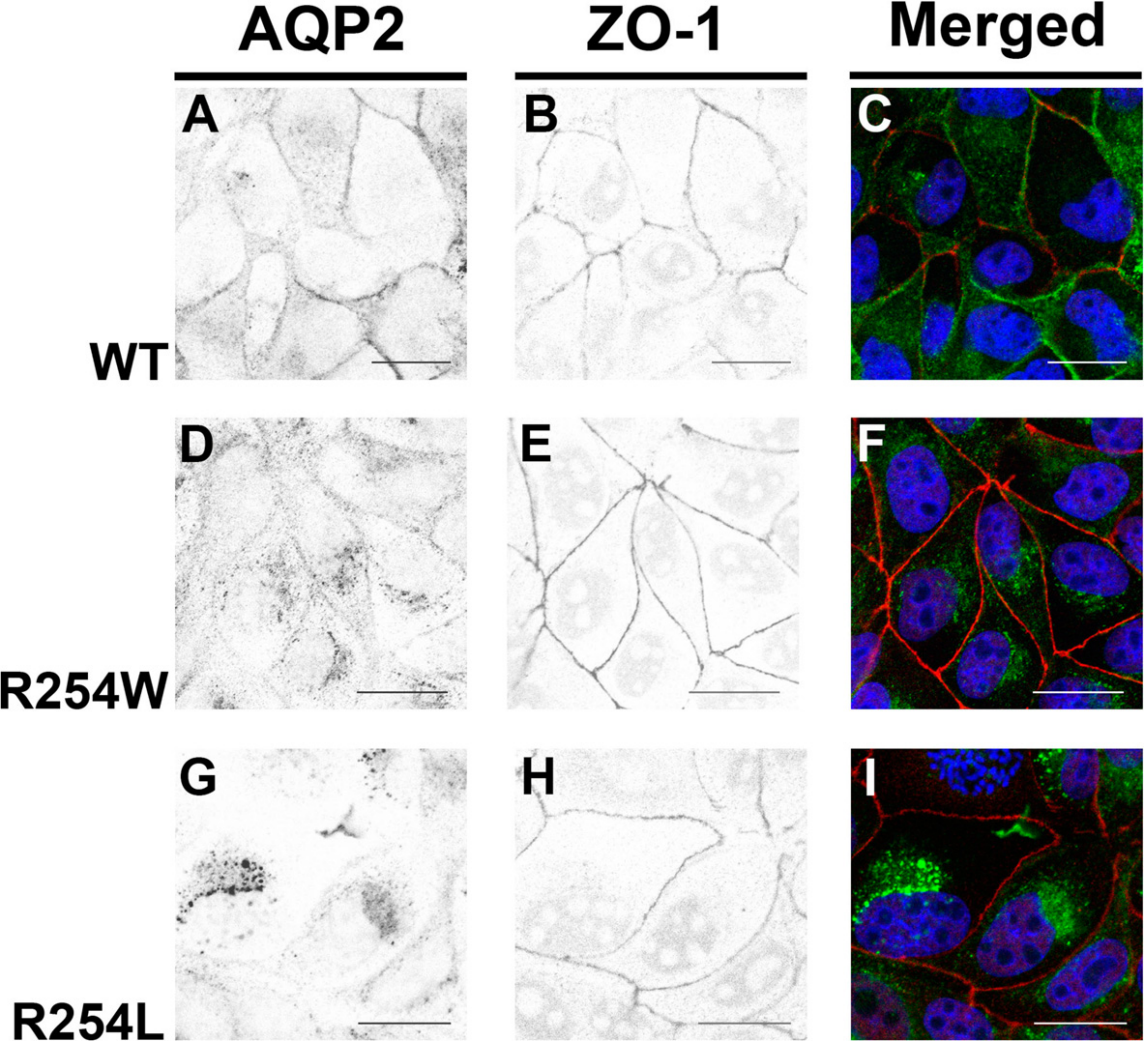
Localization analysis of AQP2-WT, AQP2-R254W and AQP2-R254L in MDCK cells under forskolin stimulated conditions. MDCK cells stably expressing AQP2-WT, AQP2-R254W or AQP2-R254L were incubated with 150 μM forskolin prior to immunostaining for AQP2 and tight junctions, ZO-1. The cells were then subjected to CLSM analysis. (**a**,**d**,**g**) Inverted contrast images of AQP2 labeling in AQP2-WT, AQP2-R254W or AQP2-R254L expressing cells. (**b**,**e**,**h**) Inverted contrast illustrations of ZO-1 expression, as detected by an anti-ZO-1 antibody, in the MDCK cells shown in **a**, **d** and **g**. (**c**,**f**,**i**) Merged color illustrations of the images shown in **a**-**b**, **d**-**e** and **g**-**h**, respectively. Scale bars = 20 μm

Having verified that the LV vector results in sustained expression and translocation of AQP2-WT from intracellular sites to the cell membrane upon forskolin stimulation, we generated by means of puromycin selection three MDCK cell lines stably expressing AQP2-WT, AQP2-R254W and AQP2-R254L proteins. Untransduced MDCK cells were used as negative controls in subsequent experiments. In contrast to AQP2-WT, almost all of the detected AQP2-R254W protein was intracellularly retained upon forskolin stimulation (compare Fig. 4a-c with 4d-f). Only very weak AQP2-specific staining at the plasma membrane was observed and the majority of AQP2-R254W protein was localized in the cytoplasm, concentrated in the perinuclear space (Fig. 4d-f). Similar results were obtained when analyzing the cellular fate of the AQP2-R254L protein (Fig. 4g-i). These observations clearly suggest that the trafficking of AQP2-R254W, like AQP2-R254L, is impaired, resulting in intracellular accumulation of variant protein.

### Phosphorylation of S256 is inhibited in AQP2-R254W

**Fig. 5:**
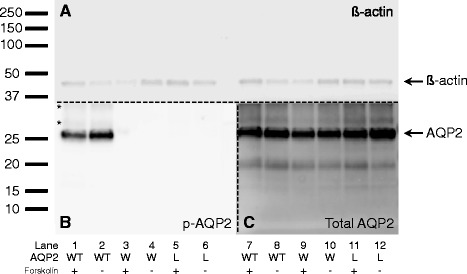
Analysis of the phosphorylation level of AQP2-WT, AQP2-R254W and AQP2-R254L in MDCK cells by means of Western blotting analysis. MDCK cells stably expressing AQP2 variant proteins were incubated with (150 μM) and without forskolin (+/-) for one hour. Following harvest and lysis, cell extract were subjected to SDS-PAGE and proteins were subsequently transferred to a PVDF membrane as described in Methods. Lanes 1, 2, 7 and 8: AQP2-WT; lanes 3, 4, 9 and 10: AQP2-R254W; lanes 5, 6, 11 and 12: AQP2-R254L. Following transfer, the membrane was cut in three separate pieces as indicated by the dotted lines and used for individual immunostaining analysis (**a**-**c**). (**a**) Loading control using an anti-ß-actin antibody. (**b**) AQP2 protein with S256 phosphorylation detected by a phosphor-sensitive anti-AQP2 antibody. The two asterisks denote p-AQP2-specific bands with an apparant higher mobility compared to the expected size of approximately 26 kDa. (**c**) Total amount of AQP2 protein as detected by an anti-AQP2 antibody. A molecular marker is indicated on the left (kDa). The presented Western blot represents the results obtained in two independent experiments

Given that the AQP2-R254W substitution disrupts a canonical and crucial PKA consensus site in AQP2 it is obvious to hypothesize that the impairment in trafficking described above is caused by impaired phosphorylation at S256 as it has been described for other R254 substitutions previously [14, 15, 25]. Thus, we investigated phosphorylation at S256 by means of Western blotting analysis using an antibody directed against S256 phophorylated AQP2 [4, 11]. Extracts from the MDCK cells (AQP2-WT, AQP2-R254W and AQP2-R254L) incubated with or without forskolin were subjected to the analysis (Fig. 5). Under both conditions the phospho-sensitive antibody detected a prominent band with an apparent mobility of approximately 26 kDa in extracts from cells expressing AQP2-WT corresponding to the expected mobility of unglycosylated AQP2 (Fig. 5b, lane 1 and 2). Even though similar levels of total AQP2-WT protein were observed with or without forskolin treatment, the level of phosphorylated AQP2 (p-AQP2) WT protein identified in unstimulated cells (Fig. 5c, lane 7 and 8) seemed higher compared to the level in forskolin stimulated cells (Fig. 5b, lane 1 and 2). Since forskolin is expected to increase phosphorylation of AQP2, this finding could reflect that the amount of detectable p-AQP2-WT in Western blotting analysis depends on the cellular localization of the protein. As shown in Fig. 3 AQP2-WT rapidly accumulates in the plasma membrane following stimulation, thereby resulting in a possible lower amount of extractable p-AQP2-WT following cell lysis, compared to the situation in unstimulated cells, in which p-AQP2-WT solely is localized intracellularly. Two faint p-AQP2-specific bands with a higher mobility, probably representing AQP2 with complex glycosylations were also recognized in the samples (indicated by asterisks in Fig. 5b, lane 1 and 2). Interestingly, no p-AQP2-specific bands could be recognized in extracts from cells expressing AQP2-R252W (Fig. 5b, lane 3 and 4) even though almost identical amounts of ß-actin (Fig. 5a) and total AQP2 protein (Fig. 5c) were present in the extracts from cells expressing AQP2-WT and AQP2-R254W, respectively. Similar results were obtained in the extracts from cells expressing AQP2-R254L (Fig. 5b, lane 5 and 6), suggesting that phosphorylation at S256 is inhibited in the AQP2-R254W protein as it is in the AQP2-R254L protein.

### Defective insertion of AQP2-R254W in the apical membrane

**Fig. 6:**
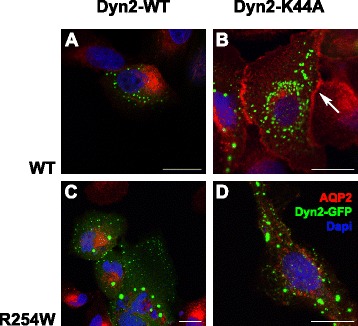
Investigation of the recycling process of AQP2 by inhibition of endocytosis via GTPase-deficient dynamin-2 mutation (K44A) in transiently transfected, unstimulated MDCK-AQP2 cells. The MDCK cells stably expressing AQP2-WT or AQP2-R254W were transiently transfected with plasmid DNA encoding GFP-tagged dynamin-2, either Dyn2-WT (**a**,**c**) or Dyn2-K44A (**b**,**d**). Two days post-transfection the cells were immunostained with AQP2-sensitive antibody and subjected to confocal laser scanning microscopy. Note the AQP2 staining of the plasma membrane in cells expressing AQP2-WT indicated with an arrow (**b**). The presented data represents the results obtained in two independent experiments. Scale bars = 20 μm

AQP2 shuttles between subapical vesicles and the plasma membrane, and phosphorylation at S256 is necessary for plasma membrane accumulation. However, when endocytosis was blocked by expression of a GTPase-deficient dynamin-2 mutant, Dyn2-K44A, both AQP2-WT and an S256 phosphorylation-deficient mutant AQP2 mutant, AQP2-S256A, accumulated in the plasma membrane in unstimulated cells [8], showing that the S256 phosphorylation is required for regulated AVP/cAMP-mediated translocation of AQP2 to the plasma membrane but not shuttling. So although AQP2-R254W could not be phosphorylated, it may still be able to shuttle to the plasma membrane. To test this, we transiently transfected either dynamin-2 WT (Dyn2-WT) or the dominant-negative Dyn2-K44A GFP-fusion-proteins into the MDCK cells stably expressing AQP2-WT and AQP2-R254W, respectively. As expected, AQP2-WT and AQP2-R254W proteins were exclusively found in intracellular vesicles upon co-expression of Dyn2-WT-GFP (Fig. 6a and c), suggesting that the majority of AQP2 is localized intracellularly at steady state in unstimulated cells. When AQP2-WT cells were co-transfected with the Dyn2-K44A-GFP construct, AQP2-WT localized to the plasma membrane in the absence of forskolin stimulation (Fig. 6b). This was in contrast to AQP2-R254W, which remained intracellularly localized despite over-expression of Dyn2-K44A-GFP with no or very low levels of AQP2-R254W observed in the plasma membrane. This indicates that the substitution of AQP2-arginine-254 to tryptophan inhibits both phosphorylation at serine-256 but also recruitment of AQP2-R254W to subapical vesicles (Fig. 6d).

### AQP2-R254W is not localized in late endosomes

**Fig. 7:**
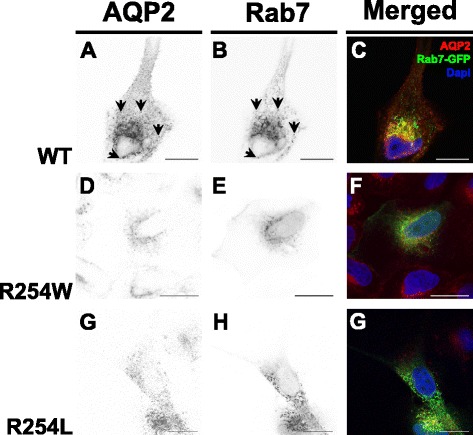
Co-localization analysis of AQP2 proteins and Rab7 positive late endosomes in unstimulated cells. MDCK cells stably expressing AQP2-WT, AQP2-R254W or AQP2-R254L were transiently transfected with plasmid DNA encoding Rab7-GFP as a marker for late endosomes. Two days post-transfection the cells were immunostained with an AQP2-sensitive antibody and subjected to confocal laser scanning microscopy. (**a**,**d**,**g**) Inverted contrast images of AQP2 labeling in AQP2-WT, AQP2-R254W or AQP2-R254L expressing cells. (**b**,**e**,**h**) Inverted contrast illustrations of the Rab7-GFP expression in the MDCK cells shown in **a**, **d** and **g**. (**c**,**f**,**g**) Merged color illustrations of the images shown in **a**-**b**, **d**-**e** and **g**-**h**, respectively. Arrows in **a** and **b** illustrates examples of co-localization of AQP2-WT and late endosomes. The presented data represents the results obtained in two independent experiments. Scale bars = 20 μm

Our presented data revealed that AQP2-R254W, similar to AQP2-R254L remains localized in intracellular vesicles upon forskolin stimulation (Figs. 3 and 4) and that the vesicles were unable to reach the plasma membrane during expression of dominant-negative dynamin 2 (Fig. 6). To determine whether these vesicles could be related to the endocytotic pathway, co-localization analysis using GFP-tagged Rab7 and Rab9, was performed. Rab7 is implicated in the transport from early to late endosomes [21], whereas Rab9 functions in the transport between late endosomes and the trans Golgi network [22]. Following transfection with plasmid DNA encoding either Rab7-GFP or Rab9-GFP fusion proteins, the AQP2-expressing MDCK cells were labeled with anti-AQP2 antibodies. AQP2 and Rab7-GFP/Rab9-GFP expression was inspected by CLSM. The majority of the AQP2-WT protein was situated in Rab7-GFP negative and Rab9-GFP negative intracellular vesicles, but some co-localization of AQP2-WT and Rab7-GFP and Rab9-GFP was observed (Fig. 7a-c and Additional file 1 A-C). Interestingly, the majority of AQP2-R254W and AQP2-R254L proteins did not co-localize with Rab7-GFP positive endosomes, suggesting that AQP2-R254W does not accumulate in endosomes to any high extent, but rather in another compartment. This indicates that AQP2-R254W may follow a different post-translational route than AQP2-WT and that the majority of AQP2-R254W does not reach sub-apical vesicles and the plasma membrane.

## Discussion

### Partial phenotype in CNDI

Clinical studies of the proband suggest that AQP2-R254W is associated with a *partial nephrogenic* DI phenotype with some ability to concentrate urine when dehydrated. This finding may thus support the notion that substitutions affecting AQP2-R254, including AQP2-R254L and AQP2-R254Q, causes partial nephrogenic DI [25]. During WDT her plasma AVP increased from 1.3 to 3.1 pg/mL as a response to the developing dehydration. Concomitantly, the urine osmolality increased significantly from 32 to 268 mOsm/kg, albeit with an endpoint slightly below 300 mOsm/kg normally used in the definition of partial DI in adults [25]. It is well known that children below 2 years of age have not yet developed a normal urine concentration capacity. The father of the proband showed an increase in urine osmolality from 126 to 613 mOsm/kg during WDT. The father was initially suspected for FNDI, which is not unusual when patients with partial CNDI are able to concentrate urine to some degree [26], and additionally respond to dDAVP in high doses. To initiate the right treatment, establishment of the correct diagnose is important. Genetic testing of patients with a family history of DI, as performed in the present study, is often a helpful tool and should therefore be considered. This is particularly important in the case of affected infants, since early diagnose might prevent dehydration episodes and related complications, such as growth retardation, cerebral damage, seizures and even death.

### AQP2-R254W causes CNDI by disabling insertion in subapical vesicles and phosphorylation at S256

The use of a phosphorylation sensitive antibody clearly demonstrated that phosphorylated AQP2 is only observed in MDCK cells expressing AQP2-WT, whereas AQP2-R254W and AQP2-R254L remained unphosphorylated in both unstimulated and forskolin stimulated cells. This indicated that phosphorylation of S256 is disabled in AQP2-R254W, similarly to AQP2-R254L. Since the R254-residue is part of the PKA consensus site (R-R_254_-X-S), substitution of arginine-254 with tryptophan most probably impairs phosphorylation at S256, similarly to what is observed for other AQP2 variations affecting the R254-residue [14, 15]. Blocking internalization with Dyn2-K44A-GFP resulted in accumulation of AQP2-WT in the plasma membrane as previously observed [27], whereas the AQP2-R254W remained localized in intracellular vesicles. This experiment demonstrated that the vast majority of AQP2-R254W does not reach the plasma membrane, and suggests that the trafficking of AQP2-R254W is affected prior to the point of sub-apical vesicle formation. As a consequence the recycling of AQP2-R254W to and from the plasma membrane is disturbed. The majority of AQP2-R254W was retained in intracellular vesicles located in the perinuclear space. Analysis of co-localization with two different late endosome markers (Rab7-GFP and Rab9-GFP) revealed some overlap with AQP2-WT, whereas AQP2-R254W and AQP2-R254L primarily co-localize with Rab7-GFP and Rab9-GFP negative endosomes, suggesting that AQP2 R254W and AQP2-R254L, in opposite to AQP2-R254Q [15] are not, or at least to a very low extend, internalized by endocytosis.

The proband and her father in this study are heterozygous for the *AQP2* variant allele, implying simultaneous synthesis of AQP2-WT and AQP2-R254W proteins in the cells of the collecting ducts. If we hypothesize that the tetramerization of AQP2 is random, some of the formed tetramers will consist of three or four AQP2-WT monomers, and hence be able to be sufficiently phosphorylated at S256 in order to undergo normal trafficking and insertion in the plasma membrane as fully functional water channels [9, 10]. This notion may thus explain their ability to concentrate the urine in response to high levels of dDAVP following medical treatment. The small percentage of published CNDI cases with autosomal dominant inheritance of the condition are all due to variations affecting the C-terminal tail of AQP2 [1], either by amino acid substitutions or by frame shift. The pathological effects of these variations seem to include defective trafficking, such as retention in the Golgi apparatus [18], misrouting of AQP2 to the basolateral membrane [16], sorting of AQP2 to the to late endosomes or lysosomes [28], or defective phosphorylation of AQP2-S256 [14], suggesting that the variations act by a dominant negative mechanism. Indeed, our findings, showing that AQP2-R254W most likely causes partial CNDI in the studied Swedish family by disabling AQP2-R254W from reaching the plasma membrane to facilitate AVP mediated urine concentration, supports these data. Furthermore, the presented data may also contribute to the understanding why the condition is usually only partial.

## Conclusions

In conclusion, this study demonstrates defective trafficking of AQP2-R254W protein to the cell surface by disabled AQP2-R254W incorporation into subapical vesicles as well as impaired phosphorylation at S256, rendering the AQP2-R254W protein predominantly to be localized in intracellular vesicles likely different from late endosomes. Studies involving pharmacological chaperones have shown some effect on retained V2 receptor variants in cells [29] and displayed beneficial effects in patients [30] whereas treatments targeting phosphorylation and trafficking defective AQP2, like AQP2-R254W, remain to be explored. The finding that the novel *AQP2* variation causes *partial* CNDI in the studied Swedish family by such a mechanism might direct studies aiming to identify new treatments in autosomal dominant CNDI.

## Additional file

Additional file 1:
**Co-localization analysis using GFP-tagged Rab9 for labeling of late endosomes in unstimulated cells.** MDCK cells stably expressing AQP2-WT, AQP2-R254W or AQP2-R254L were transiently transfected with plasmid DNA encoding Rab9-GFP as a marker for late endosomes. Two day post transfection the cells were immunostained with an AQP2-sensitive antibody and subsequently analyzed by confocal laser scanning microscopy. (A,D,G) Inverted contrast images of AQP2 labeling in cells expressing AQP2-WT, AQP2-R254W or AQP2-R254L. (B,E,H) Inverted contrast illustrations of Rab9-GFP in the MDCK cells shown in A, D and G. (C,F,G) Merged color illustrations of the images shown in A-B, D-E and G-H, respectively. Arrows in A and B illustrates examples of co-localization of AQP2-WT and late endosomes. The presented data represents the results obtained in two independent experiments. Scale bars = 20 μm. (PDF 468 kb)

## Abbreviations

AQP2: Aquaporin-2
AVP: Arginine vasopressin
CLSM: Confocal laser scanning microscopy
CNDI: Congenital nephrogenic diabetes insipidus
DAPI: 4',6-diamidino-2-phenylindole
dDAVP: 1-desamino-8-D-arginine vasopressin
DI: Diabetes insipidus
Dyn2: Dynamin-2
GFP: Green fluorescent protein
HEK: Human embryonic kidney
IRES: Internal ribosome entry site
LV: Lentivirus
MDCK: Madin-Darby canine kidney
PGK: Phosphoglycerate kinase
PKA: Protein kinase A
Puro: Puromycin
SD: Standard deviation
WTA: Water deprivation test
WT: Wild-type
