# Supplementary figures and images for: Partial nephrogenic diabetes insipidus caused by a novel AQP2 variation impairing trafficking of the aquaporin-2 water channel
Source: BMC Nephrol. 2015 Dec 29;16:217. doi: 10.1186/s12882-015-0213-3 (PMC4696136; doi:10.1186/s12882-015-0213-3)

Additional file1: Co-localization analysis using GFP-tagged Rab  
9 for labeling of late endosomes

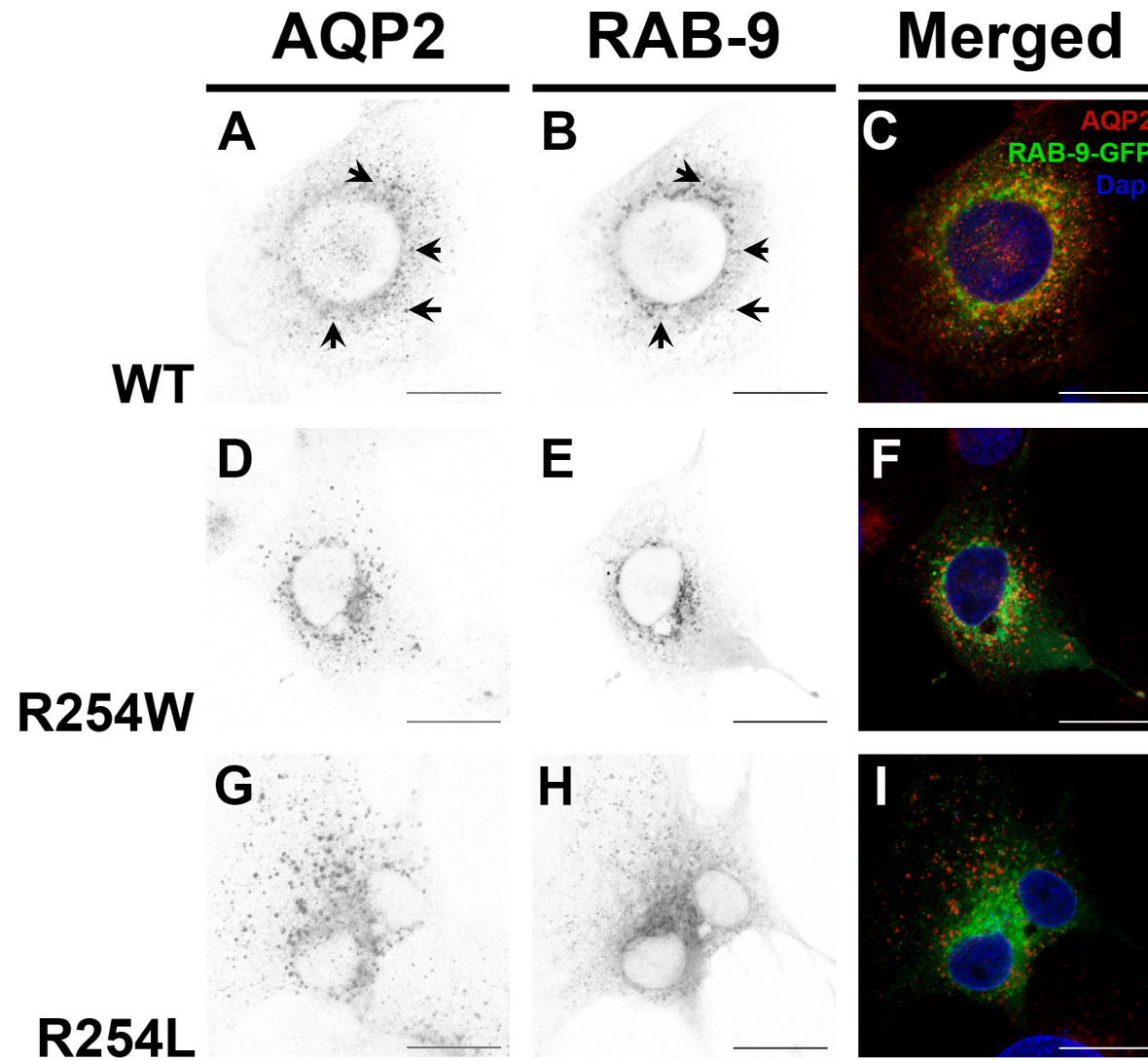

Supplement: Additional file 1: — Co-localization analysis using GFP-tagged Rab9 for labeling of late endosomes in unstimulated cells. MDCK cells stably expressing AQP2-WT, AQP2-R254W or AQP2-R254L were transiently transfected with plasmid DNA encoding Rab9-GFP as a marker for late endosomes. Two day post transfection the cells were immunostained with an AQP2-sensitive antibody and subsequently analyzed by confocal laser scanning microscopy. (A,D,G) Inverted contrast images of AQP2 labeling in cells expressing AQP2-WT, AQP2-R254W or AQP2-R254L. (B,E,H) Inverted contrast illustrations of Rab9-GFP in the MDCK cells shown in A, D and G. (C,F,G) Merged color illustrations of the images shown in A-B, D-E and G-H, respectively. Arrows in A and B illustrates examples of co-localization of AQP2-WT and late endosomes. The presented data represents the results obtained in two independent experiments. Scale bars = 20 μm. (PDF 468 kb) [file 12882_2015_213_MOESM1_ESM.pdf]
